# Interleukin 1β triggers synaptic and memory deficits in Herpes simplex virus type-1-infected mice by downregulating the expression of synaptic plasticity-related genes via the epigenetic MeCP2/HDAC4 complex

**DOI:** 10.1007/s00018-023-04817-5

**Published:** 2023-06-01

**Authors:** Domenica Donatella Li Puma, Claudia Colussi, Bruno Bandiera, Giulia Puliatti, Marco Rinaudo, Sara Cocco, Fabiola Paciello, Agnese Re, Cristian Ripoli, Giovanna De Chiara, Alessia Bertozzi, Anna Teresa Palamara, Roberto Piacentini, Claudio Grassi

**Affiliations:** 1 Department of Neuroscience, Università Cattolica del Sacro Cuore, 00168 Rome, Italy; 2 Fondazione Policlinico Universitario A. Gemelli IRCCS, 00168 Rome, Italy; 3 Department of Engineering, Istituto di Analisi dei Sistemi ed Informatica “Antonio Ruberti”, National Research Council, 00185 Rome, Italy; 4 Institute of Translational Pharmacology, National Research Council (CNR), 00133 Rome, Italy; 5 Department of Infectious Diseases, Istituto Superiore Di Sanità, 00161 Rome, Italy; 6 Department of Public Health and Infectious Diseases, Sapienza University of Rome, Laboratory Affiliated to Istituto Pasteur Italia-Cenci Bolognetti Foundation, 00185 Rome, Italy

**Keywords:** Neuroinflammation, Synaptic function, Memory, Anakinra, SUMOylation, Neurodegeneration

## Abstract

Extensive research provides evidence that neuroinflammation underlies numerous brain disorders. However, the molecular mechanisms by which inflammatory mediators determine synaptic and cognitive dysfunction occurring in neurodegenerative diseases (e.g., Alzheimer’s disease) are far from being fully understood. Here we investigated the role of interleukin 1β (IL-1β), and the molecular cascade downstream the activation of its receptor, to the synaptic dysfunction occurring in the mouse model of multiple Herpes simplex virus type-1 (HSV-1) reactivations within the brain. These mice are characterized by neuroinflammation and memory deficits associated with a progressive accumulation of neurodegenerative hallmarks (e.g., amyloid-β protein and tau hyperphosphorylation). Here we show that mice undergone two HSV-1 reactivations in the brain exhibited increased levels of IL-1β along with significant alterations of: (1) cognitive performances; (2) hippocampal long-term potentiation; (3) expression synaptic-related genes and pre- and post-synaptic proteins; (4) dendritic spine density and morphology. These effects correlated with activation of the epigenetic repressor MeCP2 that, in association with HDAC4, affected the expression of synaptic plasticity-related genes. Specifically, in response to HSV-1 infection, HDAC4 accumulated in the nucleus and promoted MeCP2 SUMOylation that is a post-translational modification critically affecting the repressive activity of MeCP2. The blockade of IL-1 receptors by the specific antagonist Anakinra prevented the MeCP2 increase and the consequent downregulation of gene expression along with rescuing structural and functional indices of neurodegeneration. Collectively, our findings provide novel mechanistic evidence on the role played by HSV-1-activated IL-1β signaling pathways in synaptic deficits leading to cognitive impairment.

**Supplementary Information:**

The online version contains supplementary material available at 10.1007/s00018-023-04817-5.

## Introduction

Accumulating evidence indicates that neuroinflammation plays a critical role in the onset and progression of many neurodegenerative and neuropsychiatric illnesses [1]. Microglial activation and overexpression of pro-inflammatory cytokines occurring in response to CNS insults may disrupt synaptic plasticity, subsequently resulting in cognitive decline [2]. Among the mediators of inflammation leading to neuronal damage, the proinflammatory cytokine interleukin-1β (IL-1β), produced and released in the brain by activated astrocytes and microglia, has been demonstrated to inhibit synaptic strength and long-term potentiation (LTP) after binding its receptor (IL-1R) that is widely expressed in the hippocampus [3, 4]. Elevated levels of IL-1β have also been detected in patients affected by memory deficits such as mild cognitive impairment and Alzheimer’s disease (AD), thus suggesting that IL-1β overproduction may be an early factor concurring to AD pathogenesis and progression [5, 6]. Despite these clues, the molecular mechanisms triggered by immune system activation and leading to synaptic dysfunction and cognitive deficits have not been fully understood yet. To get insights into the role of IL-1β in synaptic dysfunction, we took advantage of a mouse model of recurrent Herpes simplex virus type-1 (HSV-1) replication within the brain, induced by thermal stress (TS). These mice exhibited neuroinflammation (e.g., astrogliosis and elevated levels of IL-1β and IL-6) and memory deficits along with accumulation of amyloid-β protein (Aβ), and hyperphosphorylated tau (pTau) in several brain areas including hippocampus and neocortex that increased with the number of viral reactivations [7].

The protocol of HSV-1 infection and reactivation, originally set up in BALB/c mice was subsequently applied to C57BL/6 and APP knockout mice to study the effects of HSV-1 on adult hippocampal neurogenesis [7–10]. Halford and co-workers also reported that HSV-1 enters, replicates, spreads, and establishes latent infections with virtually identical efficiencies in both C57BL/6 and BALB/c mice [11]. These findings demonstrate that the effects of HSV-1 and its ability to induce an AD-like phenotype are not strain-dependent.

Here, we report that in C57BL/6 mice IL-1β negatively impinges on synapse function and structure by upregulating the expression of the epigenetic repressor methyl-CpG binding protein 2 (MeCP2), a DNA binding protein that promotes methylation-associated gene inactivation. Of note, all molecular, structural, electrophysiological, and behavioral indices of synaptic dysfunction in HSV-1-infected mice were rescued by the pharmacological blockade of the IL-1R. We also found that following HSV-1 infection the histone deacetylase 4 (HDAC4), whose alterations are correlated with synaptic dysfunction, memory impairment and inflammation [12–14], accumulates in the nucleus and promotes SUMOylation of MeCP2 thus contributing to the repression of target genes and the synaptic dysfunction observed in this mouse model of neuroinflammation.

## Materials and methods

### In vivo mouse model of recurrent HSV-1 infection

The mouse model of recurrent HSV-1 infection in the brain was established in male C57BL/6 mice as already described [7, 8]. Mock-infected mice were used as controls. For experiments involving IL-1R blockade, both mock- and HSV-1-infected mice were treated with Anakinra (30 mg/kg, i.p.) for 3 consecutive days close to each TS, specifically: the day before, the same day, and the day after TS (Fig. S1).

### Behavioral tests

Y maze, Novel Object Recognition (NOR) and Fear Conditioning (FC) paradigms were performed as in [15–17]. In the NOR test, on day one, animals were familiarized for 10 min to the test arena (45 × 45 cm). On day 2 (training session), two identical objects were placed symmetrically in the central part of the arena and each mouse was allowed to explore for 8 min. On day 3 (test session), a new object replaced one of the old objects. The animals were allowed to explore for 8 min and a preference index (PI), calculated as the ratio between time spent exploring the novel object and time spent exploring both objects, was used to measure recognition memory. Associative learning was assessed by contextual and cued FC test. On the first day (training), mice were placed in the conditioning chamber for 2 min before the onset of a discrete tone (conditioned stimulus, CS) at 2000 Hz, lasting for 30 s. In the last 2 s of the CS, mice were given a foot shock (unconditioned stimulus, US) of 0.70 mA through metal floor bars. After the CS/US pairing, mice were left in the conditioning chamber for 30 s and then they were placed back in their home cages. On the second day, the contextual fear learning was evaluated: mice were placed back in the conditioning chamber, without CS presentation, and their freezing behavior, defined as the absence of all movements except for that necessitated by breathing, was measured for 5 consecutive min. On the third day, cued fear learning was evaluated: mice were placed in a novel context for 2 min (pre-cued) and then they were exposed to the CS for 3 min (cued), and freezing was measured. Both tests were recorded and analyzed using ANY-maze (Stoelthing, Wood Dale, IL, USA) by an experimenter blind to treatments. For the evaluation of spatial working memory we employed the Y-maze test. Briefly, the animals were allowed to explore the 3 arms of a Y-shaped apparatus for 8 min made of non-transparent white plexiglas. The number and order of entrances within the 3 different arms were recorded. Spontaneous alternations behavior was then quantified as percentage of the number of correct triplets, defined as sequential entrances within the 3 different arms divided by the number of total entrances minus 2. A higher percentage of spontaneous alternations reflects an intact spatial working memory ability. Between each animal, the apparatus was cleaned using a 70% ethanol solution.

### Electrophysiological recordings

LTP recordings were performed on 400 μm-thick hippocampal coronal slices from mock- and HSV-1-infected 4-month-old mice as in [18, 19]. To study LTP in CA3-CA1 synapses, field excitatory postsynaptic potentials (fEPSPs) were recorded in the CA1 stratum radiatum by a glass capillary filled with artificial cerebrospinal fluid in response to stimulation of the Schaffer collaterals by a bipolar tungsten electrode. First, the input–output relationship was constructed and the stimulation intensity that elicited one-third of the maximal response was used for delivering test pulses every 20 s. After achieving a stable baseline response, LTP was induced by using the High Frequency Stimulation (HFS) protocol (one train of stimuli at 100 Hz, lasting 1 s, repeated four times with an inter-train interval of 10 s). LTP magnitude (measured at 55–60 min after induction) was expressed as the percentage change in the mean fEPSP peak amplitude normalized to baseline values (i.e., mean values for the last 10 min of recording before HFS). Recordings were performed in current clamp I = 0 mode, using a Multiclamp 700B/Digidata 1440 B system (Molecular Devices, San Jose, CA, USA).

### Western blot

Western blot (WB) analysis was performed on hippocampal tissues as in [9, 10, 20–23]. Equal amounts of proteins (30–50 μg) were diluted in Laemmli buffer, boiled, and resolved by SDS-PAGE. The primary antibodies (Table S1) were incubated overnight at 4 °C and revealed with HRP-conjugated secondary antibodies (Cell Signaling Technology Inc. Danver, MA). Protein expression was evaluated by using UVItec Cambridge Alliance. Molecular weights were identified using precision Plus Protein Dual Color Standards (Bio-Rad, Hercules, CA, USA). Data are reported as “fold induction” vs. control. WB experiments were performed in four independent replicates. The total number of samples analyzed (number of mice) for condition is reported in the figure legends.

### mRNA isolation and first strand cDNA synthesis

Hippocampal tissues from mock- and infected animals treated and untreated with Anakinra were homogenized in Trizol (Thermo Fisher Scientific, Waltham. MA, USA) and total RNA was isolated according to the manufacturer's instructions. cDNA was synthesized using a High-capacity cDNA reverse transcription kit (Applied Biosystems, Foster City, CA, USA) for real time PCR or using RT^2^ PCR Array First Strand Kit (Qiagen, Hilden, Germany) to perform RT^2^ Profiler PCR Array. See details in Supplementary Material and Methods.

### Real-time quantitative PCR

Quantitative real-time PCR amplifications were performed as described in [8] using specific primers (Table S2) and the Power SYBR™ Green PCR Master Mix (Applied Biosystems) on AB7500 instrument. Samples were run in triplicate and relative mRNA levels for genes of interest were normalized to glyceraldehyde 3-phosphate dehydrogenase and rRNA 18S and calculated by using the 2^−ΔΔCt^ method.

### Immunohistochemistry

Hippocampal slices were processed for immunofluorescence (IF) as in [8]. See Supplementary Material and Methods for details.

### Golgi–Cox staining for spine density and morphology

Immunocytochemistry and morphological spine classification were carried out as in [24]. The number of dendritic spines was counted in blind in both apical and basal dendrites of CA1 pyramidal neurons. Images were captured through a 100 × oil-immersion objective and analyzed by Image J (NIH).

### ELISA assay

Murine IL-1β concentration was determined by ELISA kit (Immunological Sciences, Rome, Italy) according to the manufacturer’s instructions**.**

### Co-Immunoprecipitation

Co-Immunoprecipitation (Co-IP) experiments and the relative association between proteins were performed as in [25]. Brain-isolated hippocampi were lyzed in RIPA buffer (10 mM Tris–HCl (pH 7.4), 140 mM NaCl, 1% tritonX100, 0.1% sodium deoxycholate, 0.1% SDS, 1 mM EDTA) supplemented with 1 mM PMSF and protease inhibitor cocktail. Co-IP experiments were performed using 3 μg of antibody for 400 μg of protein extract. Immune complexes were purified using the Ademtech's Bio-Adembeads paramagnetic bead technology. Equal amounts of protein extract samples were immunoprecipitated with the corresponding purified IgG and used as a negative control (Santa Cruz; Dallas, Texas, USA). Total sample inputs were run in the WB as loading controls. In Co-IP experiments, the relative association between proteins was performed calculating the band OD ratio between the target proteins and the bait (HDAC4).

### SUMOylation assay

Samples were extracted in RIPA buffer containing 1% SDS plus a protease inhibitor mix and 50 mM N-Ethylmaleimide (NEM) to block SUMO proteases. Lysates (500 µg of total protein) were subjected to immunoprecipitation using anti-MeCP2 antibody (3 µg/sample) crosslinked to paramagnetic beads (Bioadembeads) over-night. The immune complexes were washed and eluted according to the manufacturer’s instruction and used in WB experiment to detect SUMO-1ylation using anti-SUMO-1 antibody and the amount of bait (MeCP2) immunoprecipitated with anti-MeCP2. SUMO-1-MeCP2 was identified as molecular bands above 75 KDa running slower than the unmodified protein. The ratio of OD values from SUMO-1 and MeCP2 signals was used to calculate the SUMOylation index.

### Nuclear-cytoplasmic fractioning

Nuclear and cytosolic fractions were prepared from hippocampi with the NE-PER kit (NE-PER ThermoFisher) according to manufacturer’s instructions.

### Chromatin immunoprecipitation

Chromatin immunoprecipitation (ChIP) assays were carried out as in [26]. Tissues were resuspended in 200 μl lysis buffer containing 1% SDS, 50 mM Tris–HCl pH 8.0, and 10 mM EDTA and sonicated on ice with six 10-s pulses with a 20-s interpulse interval. Sample debris was removed by centrifugation and supernatants were precleared using protein G-Sepharose 4B beads (Sigma-Aldrich) for 1 h at 4 °C. Two μg of HDAC4 antibody were added overnight at 4 °C. Immune complexes were collected by incubation with protein G-Sepharose 4B beads for 2 h at 4 °C. After seven washes, immunoprecipitated complexes were isolated from beads by vortexing in 150 μL of elution buffer (1% SDS and NaHCO3 0.1 M; pH 8.0). After addition of NaCl, lysates were incubated overnight at 65 °C to reverse protein-DNA cross-linking. PCR DNA fragments purification kit was used to recover chromatin fragments (Geneaid Biotech Ltd, Xizhi District, New Taipei City, Taiwan). PCR conditions and cycle numbers were determined empirically, and each PCR reaction was performed in duplicate. Data are expressed as percentage of input calculated by the “Adjusted input value” method according to the manufacturer’s instructions (Thermo Fisher Scientific ChIP analysis). The C_t_ value of the input was subtracted by 6.644 to calculate the adjusted input (i.e., log2 of 100). The percent input of control and IP samples was then computed using the following formula: 100 × 2 (adjusted input − C_t_ (ChIP)). The percent input of the No-antibody samples was calculated using the formula 100 × 2 (adjusted input − C_t_ (NoAb)). Primers used for promoter analysis are indicated in Table S2.

### Statistical analysis

Statistical significance was assessed by using the appropriate statistical analysis for the various comparisons. The equal variance between groups and normal distribution of data was checked by the Shapiro–Wilk test before performing statistical analysis. The statistical tests used (i.e., Student’s t test, Mann–Whitney Rank Sum Test, two-way ANOVA) are indicated in the main text and in the corresponding figure legends for each experiment. The number of repetitions (n) for each experimental condition is reported in the figure legends.

Two-way ANOVA test with Student–Newman–Keuls post-hoc correction was used to compare multiple groups, whereas for experiments that included fewer than 10 observations, or for non-normally distributed data, the Mann–Whitney Rank Sum Test or Kruskal–Wallis One Way Analysis of Variance on Ranks were used. Data were expressed as mean ± SEM. The level of significance was set at p less than 0.05. Statistical comparisons and analyses were carried out with SigmaPlot software 14.0.

## Results

### HSV-1-induced increases of IL-1β impair hippocampal-dependent memory in mice

**Fig. 1:**
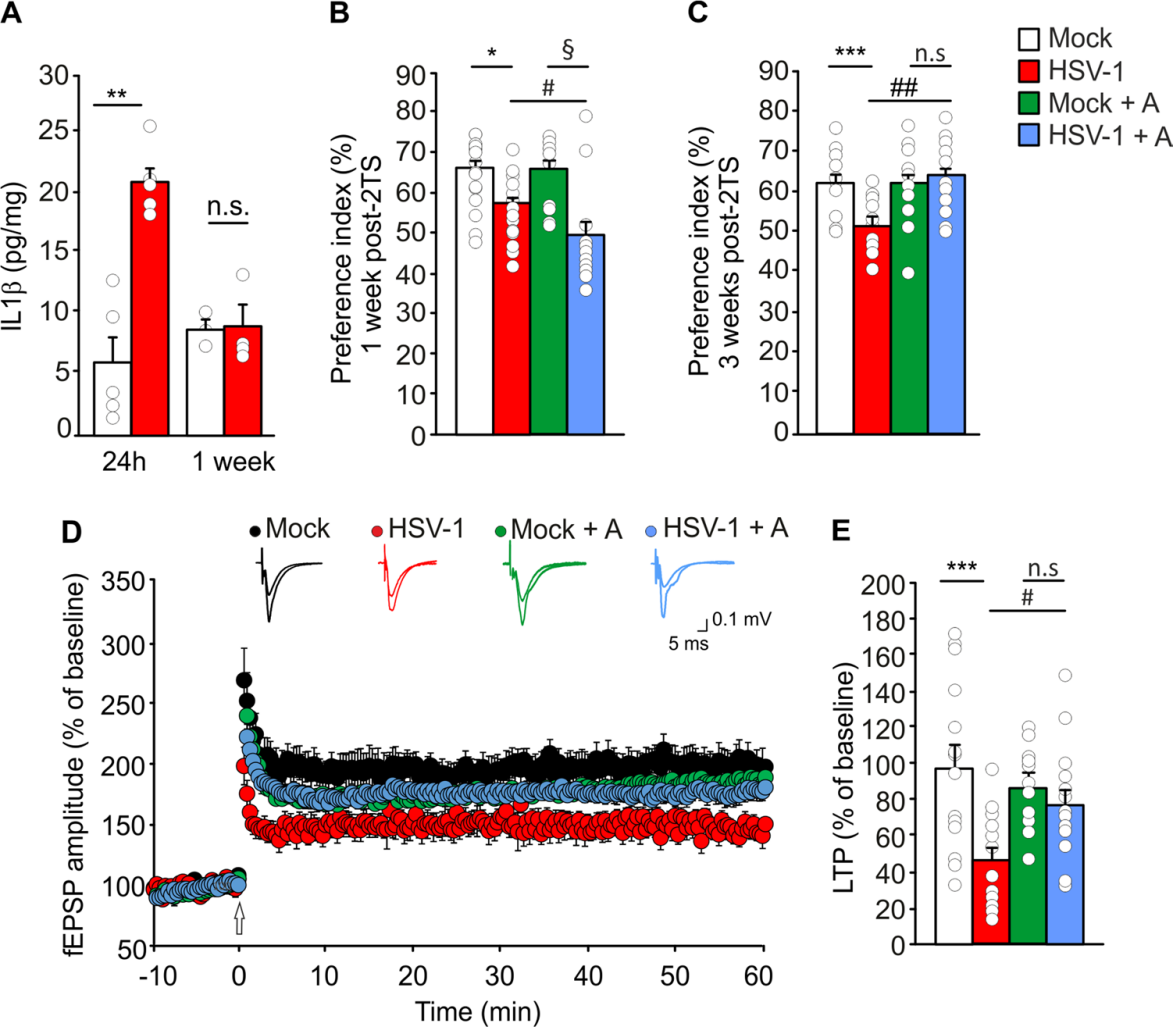
Inhibition of IL-1R restores LTP and hippocampal-dependent memory in HSV-1-infected mice undergoing 2TS. **A** Bar graph showing IL-1β levels in hippocampal homogenates from mock- and HSV-1-infected mice sacrificed 24 h (n = 5 for each group) and 1 week (n = 3 and 4, respectively) after 2TS; **B**, **C** Bar graphs showing the mean values of PI for the novel object in NOR test performed: **B** 1 week after 2TS (n = 16 mock, n = 15 HSV-1, n = 10 mock + A and n = 12 HSV-1 + A); **C** 3 weeks after 2TS in mock- and HSV-1-infected mice treated and untreated with Anakinra (n = 13 mice/group); **D** mean time course of fEPSP amplitude before and after high-frequency stimulation (indicated by arrow) in hippocampal slices from: mock- (n = 15 slices from 7 mice) and HSV-1-infected mice (n = 15 slices from 6 mice) and mice treated with Anakinra [mock + A (n = 11 slices from 8 mice) and HSV-1 + A (n = 15 slices from 7 mice)] sacrificed 1 week afteṙ 2TS; **E** Bar graphs showing mean LTP in the last 5 min of recording in controls and infected mice. *p < 0.05, **p < 0.01 and ***p < 0.005 vs. mock; ^§^p < 0.05 vs. mock + A; ^#^p < 0.05 and ^##^p < 0.005 vs. HSV-1 assessed by Mann–Whitney Rank Sum Test (**A**) and by two-way ANOVA followed by Student–Newman–Keuls post-hoc (**B**–**E**). *n.s.* not significant difference

We first checked whether repeated HSV-1 reactivations in the brain of C57BL/6 mice, already demonstrated to be vulnerable to hippocampal HSV-1 infection [11], triggered neuroinflammation by focusing on the pro-inflammatory cytokine IL-1β, whose elevated levels have been reported to impair neuronal plasticity and memory [27]. Hippocampal tissues of HSV-1-infected mice analyzed 24 h after the 2nd TS (2TS) exhibited increased levels of IL-1β, evaluated by ELISA assay, with respect to mock-infected ones (20.9 ± 1.1 vs. 5.8 ± 2.1 pg/mg, respectively; p = 7.9 × 10^–4^), that returned to control values one week later (8.8 ± 0.8 vs. 9.2 ± 1.6 pg/mg in mock- and HSV-1-infected mice, respectively; p = 0.63; Fig. 1A).

We previously reported in a different mouse strain (BALB/c) that HSV-1 infection induced impairment of cognitive functions but the underlying molecular mechanisms were not investigated [7]. To address the link between increased levels of IL-1β and cognitive dysfunction in our experimental model we studied the effects of HSV-1 infection on cognitive function of C57BL/6 mice by an array of behavioral tests assessing recognition, spatial working, and associative memory, i.e., NOR, Y-maze and FC paradigms, respectively. By looking at recognition memory, one week after 2TS the preference index (PI) for the novel object was significantly decreased in infected mice compared to controls (57.1 ± 2.1 vs. 65.8 ± 2.1, respectively; p = 1.2 × 10^–3^; Fig. 1B), and this difference was still significant three weeks later (52.2 ± 1.8 vs. 62.0 ± 2.3, respectively; p = 4.1 × 10^–4^; Fig. 1C). Infected mice also exhibited altered spatial working and associative memory, as demonstrated by a reduced percentage of spontaneous alterations in Y-maze test (59.4 ± 2.9 vs. 70.5 ± 2.6, p = 6.5 × 10^–4^; Fig. S2A), as well as a lesser freezing behavior (% time) in the contextual FC paradigm (30.5 ± 3.0 vs. 41.9 ± 3.1; p = 1.1 × 10^–3^; Fig. S2B) with respect to mock-infected ones. Instead, amygdala-dependent memory, assessed in the same mice 24 h after contextual FC, was not affected (Fig. S2C). Looking for a correlation between the increased hippocampal levels of IL-1β and memory impairment observed in HSV-1-infected mice, we treated animals with Anakinra, a pharmacological inhibitor of IL-1R, already used to treat chronic or acute inflammatory diseases [28, 29]. The efficacious dose of Anakinra in C57BL/6 mice (30 mg/kg) was identified by studying its ability to counteract the IL-1β-dependent increase of the inducible nitric oxide synthase (iNOS, [30]) observed in the brain of infected mice (+ 128% with respect to controls; p = 4.0 × 10^–3^; Fig. S3A, B). Before studying the effects of Anakinra on memory of HSV-1-infected mice, we tested whether this drug inhibited viral replication by evaluating the virus titer in the supernatants of infected cultured murine neurons in the presence or not of Anakinra (1.49 mg/ml) that were 2.4 × 10^2^ and 1.5 × 10^2^ plaque forming units/ml, respectively, 24 h post infection (p > 0.05, data not shown).

Recognition memory was not ameliorated by Anakinra treatment one week after 2TS (49.3 ± 3.9 vs. 66.0 ± 2.5, p = 2.3 × 10^–5^ in HSV-1- and mock-infected mice, respectively; Fig. 1B) but it was significantly improved three weeks later. At this time point, the PI of infected mice treated with Anakinra was significantly higher than in HSV-1 mice untreated with the IL-1R blocker (63.0 ± 2.7; vs. 52.2 ± 1.8, p = 1.8 × 10^–4^) and it was not significantly different from that of mock-infected mice treated with Anakinra only (60.4 ± 3.0; p = 0.45; Fig. 1C).

### HSV-1 infected mice exhibit structural and functional synaptic defects induced by IL-1β

**Fig. 2:**
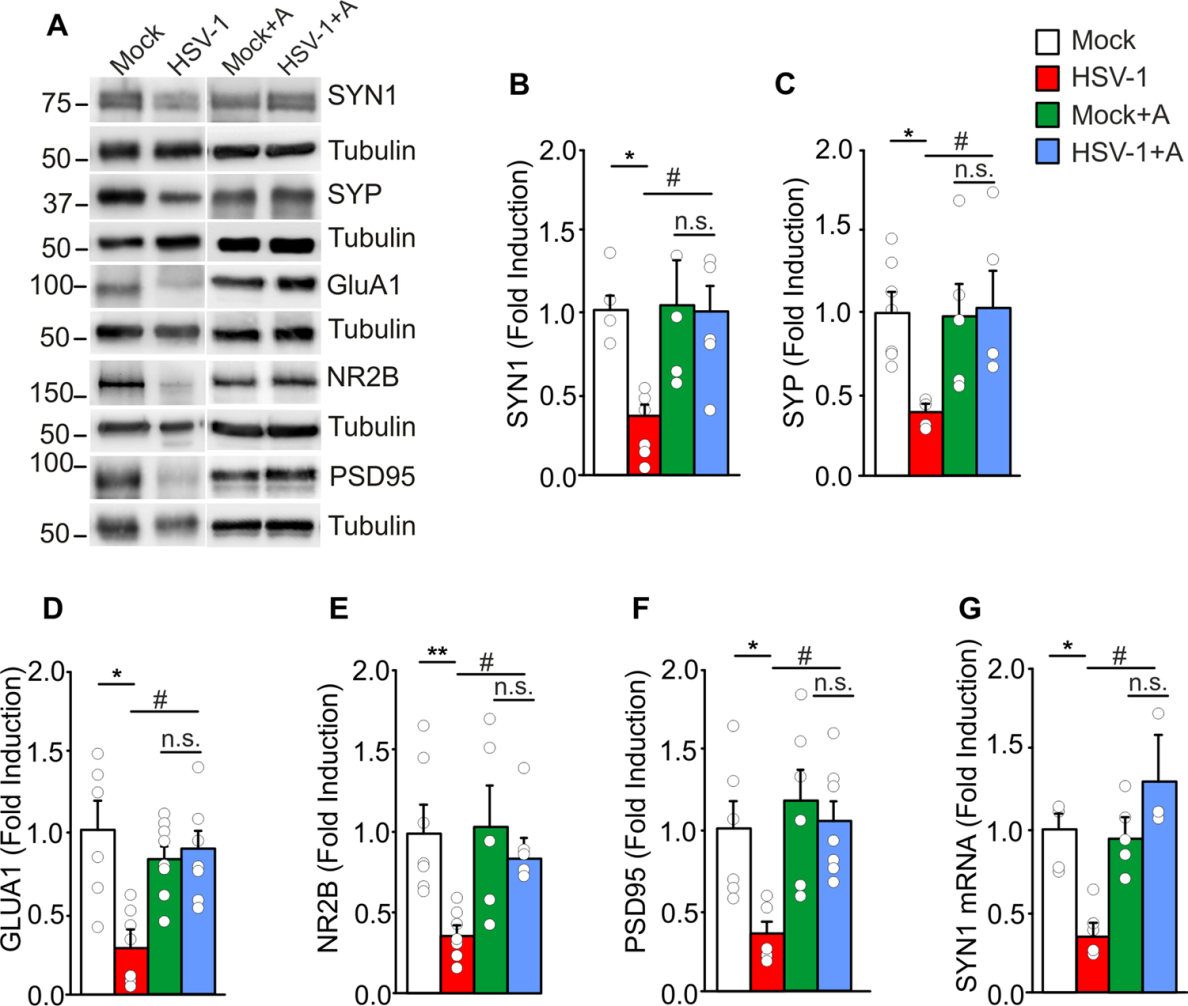
Anakinra rescues synaptic deficits induced by IL-1β in HSV-1-infected mice. **A** Representative WB images of pre- and post-synaptic proteins in hippocampal lysates from mock- and HSV-1-infected mice treated or untreated with Anakinra; **B**, **C** Bar graphs showing the densitometric analysis of SYN1 (**B**) and SYP (**C**) in mock- (n = 6 and n = 7, respectively) and HSV-1-infected mice (n = 6 and n = 5), and in Anakinra-treated mock- (n = 6 and n = 5) and infected mice (n = 6 and n = 5); **D**–**F** Densitometric analysis of postsynaptic proteins: **D** GluA1 (n = 6 mock, n = 7 HSV-1, n = 9 mock + A and n = 8 HSV-1 + A); **E** NR2B (n = 7 for both mock- and HSV-1-infected mice, n = 5 mock + A, n = 6 HSV-1 + A); and **F** PSD95 (n = 6 mock, n = 7 HSV-1, n = 6 mock + A and n = 9 HSV-1 + A); **G** Relative expression of mRNA encoding for SYN1 (n = 5 mock, n = 6 HSV-1 infected mice, n = 5 Anakinra-treated controls and n = 3 Anakinra-treated HSV-1-infected mice). *p < 0.05, **p < 0.01 and vs. mock, ^#^p < 0.05 vs. HSV-1-infected mice assessed by Kruskal–Wallis One Way Analysis of Variance on Ranks. *n.s.* not significant difference

To get insights on the detrimental effects of HSV-1 infection on memory, we studied LTP at CA3-CA1 hippocampal synapse that is considered the cellular correlate of learning and memory [31]. In brain slices obtained from HSV-1-infected mice one week after 2TS, synaptic plasticity was significantly lower than in those from mock-infected mice, with LTP being 47.3 ± 6.4% vs. 98.0 ± 12.4% greater than baseline, respectively (p = 2.1 × 10^–5^ vs. mock-infected ones; Fig. 1D, E). Hippocampal LTP deficits in infected mice were also associated with a significant downregulation of genes related to synaptic functions. By using the mouse Synaptic Plasticity RT^2^ Profiler PCR Array, we analyzed the expression of a panel of genes critical for LTP and long-term depression, the expression of neuronal receptors and synaptic remodeling. Out of 84 genes analyzed, 6 immediate early genes (ARC, EGR1, EGR2, FOS, NR4A1, PIM1) and 2 postsynaptic density regulatory genes (ADAM10, ARC) were significantly decreased in hippocampal tissue of infected mice undergoing 2TS (Table 1). Given that synaptic plasticity relies on molecular and structural changes occurring at the synapse [32], we further characterized our mouse model of HSV-1 brain infection in term of synaptic protein expression and dendritic spine density. WB analyses revealed a significant reduction of synapsin-1 (SYN1) and synaptophysin (SYP) expression in hippocampal tissue from HSV-1-infected mice [− 64% (SYN1), p = 2.0 × 10^–3^ and − 61%, (SYP), p = 1.2 × 10^–3^ vs. mock-infected ones; Fig. 2A–C]. Similar results were obtained with IF experiments (− 49% and − 41%, p = 2.2 × 10^–4^ and p = 2.1 × 10^–4^ vs. mock for SYN1 and SYP, respectively; Fig. S2D-M]. The effects of viral infection and reactivation on the post-synaptic protein levels were assessed by studying the expression of GluA1 subunit of the α-amino-3-hydroxy-5-methyl-4-isoxazolepropionic acid receptor (− 71%), NR2B subunit of N-methyl-d-aspartate receptors (− 64%) and PSD95 (− 64%, p = 1.0 × 10^–3^ and p = 6.0 × 10^–4^, p = 3.3 × 10^–3^, respectively, vs. mock-infected mice Fig. 2A, D–F).

**Table 1:** Recurrent HSV-1 infection downregulates synaptic plasticity-related genes

Synaptic plasticity genes	Fold change	p value
ADAM10^a,b,c^	− 2.30	0.04
ARC^c,d^	− 6.56	0.0009
EGR1^d^	− 3.64	0.006
EGR2^d^	− 19.81	0.036
FOS^d^	− 11.69	0.035
NR4A1^d^	− 3.83	0.007
PIM1^d^	− 3.35	0.008

Mouse synaptic plasticity genes were downregulated in HSV-1 infected mice undergone 2TS: of out of 84 genes analyzed, categorized in 10 groups, only the genes significantly downregulated were shown in this table. p < 0.05 vs. mock-infected mice

^a^Cell adhesion

^b^Extracellular matrix and proteolytic processing

^c^Postsynaptic density

^d^Immediate-early response genes (IEGs)

**Fig. 3̇:**
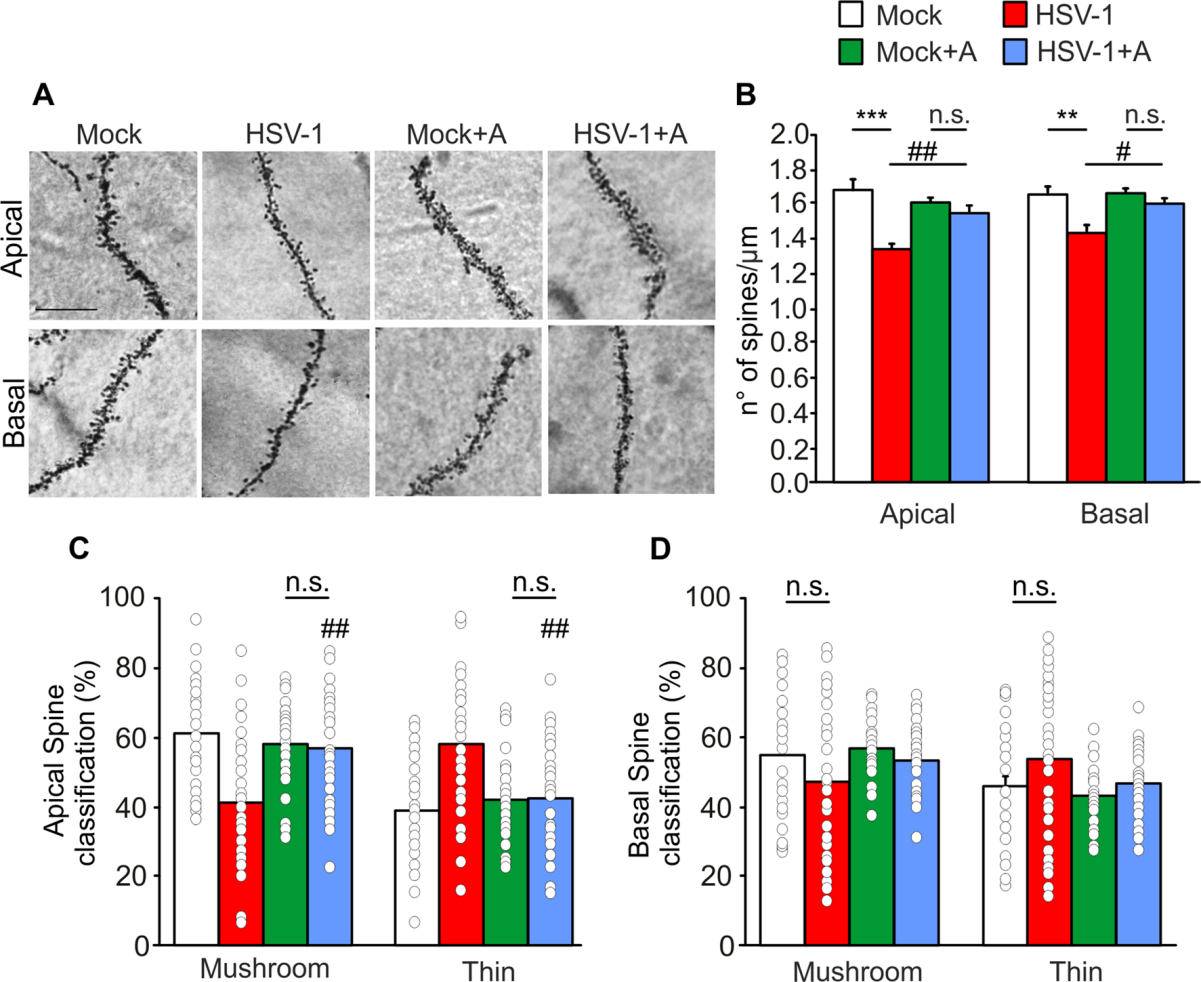
Dendritic spine density and morphology are restored in Anakinra treated HSV-1-infected mice. **A** Representative images of Golgi staining of apical and basal dendrites of CA1 pyramidal neurons of mock- and HSV-1-infected animals with and without Anakinra treatment (n = 4 mice/group, at least 10 neurons/animal); **B** Bar graph showing mean values of spine density in all neurons examined in **A**; **C**, **D** Bar graphs showing the percentage of mushroom and thin spine types of apical (**C**) and basal (**D**) dendrites in Anakinra treated and untreated mice. **p < 0.01 and ***p < 0.005 vs. mock, ^#^p < 0.05 and ^##^p < 0.005 vs. HSV-1, assessed by two-way ANOVA and Newman-Keuls post-hoc. *n.s.* not significant difference. Scale bar 10 µm

Finally, we examined dendritic spine number and morphology of hippocampal CA1 pyramidal neurons. We found a significant reduction of spine density both in apical dendrites (from 1.67 ± 0.06/µm of dendrite length in the mock to 1.34 ± 0.03/µm in HSV-1-infected mice; p = 9.0 × 10^–7^) and in basal ones (from 1.64 ± 0.06 to 1.43 ± 0.04/µm in mock- and HSV-1-infected mice, respectively; p = 9.3 × 10^–4^; Fig. 3A, B). This reduction was associated with loss of spine maturation in apical dendrites of infected CA1 pyramidal neurons, as highlighted by a decrement in the percentage of mushroom-shaped spines (from 61 ± 3 to 42 ± 4% of the total spines, p = 2.0 × 10^–6^ vs. mock), paralleled by a significant increment of immature, thin-type spines (from 39 ± 3 to 58 ± 4% of the total spines, p = 2.0 × 10^–6^ vs. mock; Fig. 3C). Instead, no significant differences were found in basal dendrite spine morphology (p = 0.08 for both mushroom and thin-type spines, Fig. 3D).

In line with what we observed in behavioral experiments, Anakinra treatment prevented all the functional, molecular and structural detrimental effects observed in our model. Indeed, no significant differences were found between mock- and HSV-1-infected mice treated with Anakinra on: (1) fEPSP potentiation (86.7 ± 7.3% vs. 77.5 ± 8.2%, respectively, p = 0.48; Fig. 1D, E); (2) expression profile of synaptic plasticity-related genes (Table 2 and Fig. 2G) and pre- and post-synaptic protein levels (p > 0.05 vs. mock + Anakinra; Fig. 2A–F); (3) spine density at apical and basal dendrites (p = 0.31 and p = 0.62, respectively, vs. mock + Anakinra; Fig. 3A, B) and their morphology (p = 0.93 vs. Anakinra-treated mock-infected mice and p = 8.5 × 10^–6^ vs untreated HSV-1-infected mice for mushroom and thin-type spines, Fig. 3D).

**Table 2:** Inhibition of IL-1R rescues synaptic genes downregulated in HSV-1-infected mice

Synaptic plasticity genes	Fold change	p value
ADAM10^a,b,c^	− 1.63	0.11
ARC^c,d^	1.30	0.87
EGR1^d^	− 1.13	0.59
EGR2^d^	1.35	0.78
FOS^d^	1.96	0.59
NR4A1^d^	− 1.61	0.44
PIM1^d^	− 1.84	0.41

Mouse synaptic plasticity genes, downregulated in HSV-1-infected mice, were significantly rescued in HSV-1-infected mice treated with Anakinra. p > 0.05 vs. mock-infected mice treated with Anakinra

^a^Cell adhesion

^b^Extracellular matrix and proteolytic processing

^c^Postsynaptic density

^d^Immediate-early response genes (IEGs)

### IL-1β upregulates MeCP2 expression in HSV-1-infected mice

**Fig. 4:**
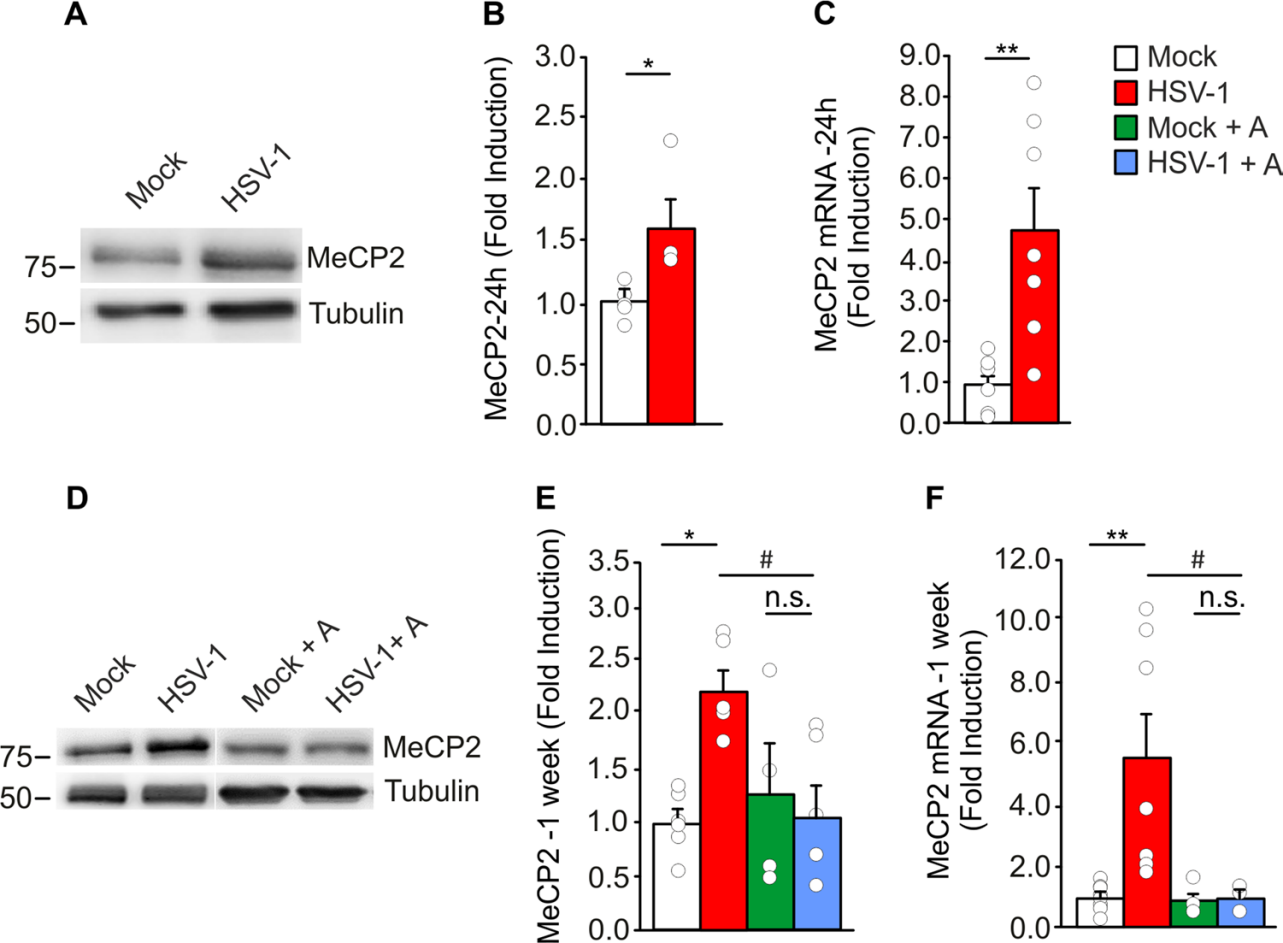
IL-1β upregulates the epigenetic repressor MeCP2 in HSV-1-infected mice. **A**, **B** Representative images (**A**) and quantification (**B**) of WB analysis for MeCP2 protein levels in hippocampal homogenates from mock- (n = 4) and HSV-1-infected mice (n = 4) sacrificed 24 h after 2TS; **D**, **E** Representative WB images (**D**) and quantification (**E**) of MeCP2 protein levels in untreated mock- (n = 6) and HSV-1-infected mice (n = 6) and in animals treated with Anakinra (n = 4 for mock; n = 6 for HSV-1-infected mice analyzed 1 week after 2TS; **C**, **F** Relative expression of mRNA encoding for MeCP2 (n = 7 for both) analyzed 24 h (**C**) and 1 week (**F**) after 2TS (n = 8 for untreated control mice and n = 7 for untreated infected ones; n = 4 and n = 3 for Anakinra-treated controls and HSV-1-infected mice). Tubulin was used as a loading control. *p < 0.05, **p < 0.01 vs. mock, ^#^p < 0.05 vs. HSV-1, assessed by Mann–Whitney Rank Sum Test (for **B**, **C**) and Kruskal–Wallis One Way Analysis of Variance on Ranks (for **E**, **F**). *n.s.* not significant difference

To investigate the causal link between increased levels of IL-1β and synaptic dysregulation observed in HSV-1 mice, we focused our attention on the epigenetic repressor MeCP2 which has been reported to bind the promoters of numerous neural genes, including those coding for the vast majority of synaptic proteins. Activation of MeCP2 can promote the methylation of target genes thus explaining the synaptic impairment caused by neuroinflammation [33, 34]. We found increased levels of MeCP2 24 h after 2TS in terms of both protein (+ 59% vs. mock, p = 2.8 × 10^–3^; Fig. 4A, B) and mRNA expression (+ 4.8 folds vs. mock, p = 6.9 × 10^–4^; Fig. 4C). Interestingly, both protein and mRNA levels of MeCP2 were still significantly higher than controls (+ 128 and + 5.5 folds, respectively vs. mock; p = 4.6 × 10^–3^ and p = 6.0 × 10^–4^; Fig. 4D–F) in infected mice sacrificed 1 week after 2TS, when IL-1β levels had returned to normal values. The HSV-1-induced upregulation of the repressor was mediated by IL-1β, as suggested by data obtained following IL-1R antagonist treatment. Indeed, the levels of MeCP2 protein and mRNA in HSV-1-infected mice treated with 30 mg/kg Anakinra, were not significantly different from those of mock-infected mice (Fig. 4D–F).

### HDAC4 is part of a new MeCP2-containing molecular complex

**Fig. 5:**
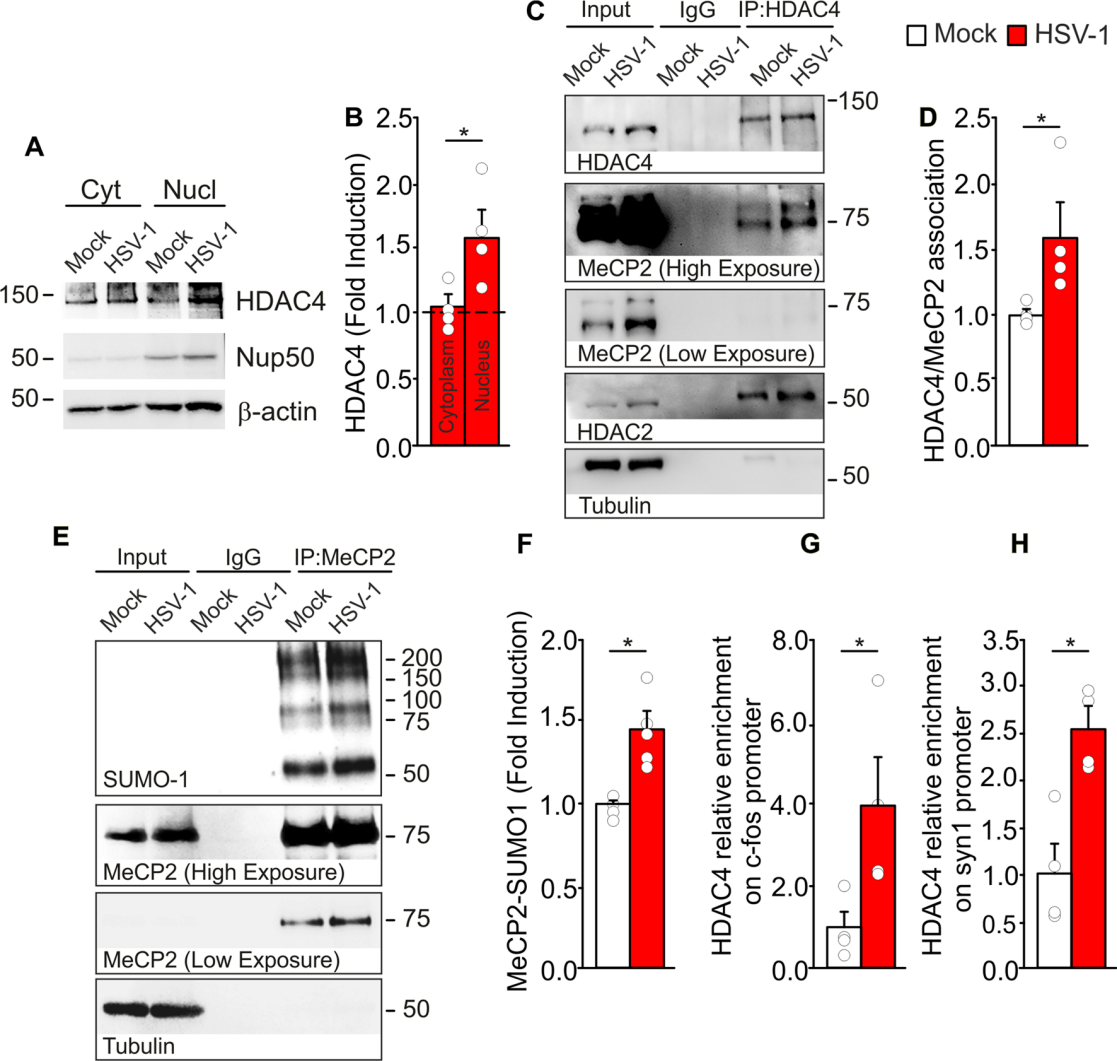
In HSV-1-infected mice nuclear HDAC4 interacts with MeCP2 and promotes its SUMOylation. **A**, **B** Cellular fractioning showing HDAC4 distribution in the nuclear and cytoplasmic compartments in hippocampal extracts from mock- and HSV-1-infected mice and their densitometric analyses (n = 4 mice/group). Dotted line indicates the cytoplasmic and nuclear levels of HDAC4 in the controls; **C** representative Co-IP experiment showing HDAC4 interaction with MeCP2 in hippocampal tissues form mock- and HSV-1-infected mice (n = 4 mice/group) and its quantification (**D**); HDAC2 was used as control of interaction; **E**, **F** Co-IP showing SUMO-1-MeCP2 levels in mock- and HSV-1-infected mice (n = 5 mice/group) and its quantification; **G**, **H** ChIP assay showing HDAC4 recruitment on *c-fos* and *syn1* promoters performed on HSV-1- and mock-infected animals (n = 4 mice/group). *p < 0.05 vs. mock, assessed by Mann–Whitney Rank Sum Test

To understand how synaptic plasticity genes were negatively regulated by MeCP2 under aberrant IL-1β signaling, we investigated the components of the regulatory machinery focusing our attention on HDACs that are important co-repressors in MeCP2-containing complexes [35, 36]. Among HDACs, HDAC4 has gained much attention since its dysfunction and nuclear accumulation occurring in AD and in experimental model of neurodegeneration contribute to synaptic dysfunction, memory impairment and inflammation through the repression of several synaptic genes and/or by post-translational modifications (PTMs) [12–14]. First, to check the presence of HDAC4 alterations induced by HSV-1 we performed a subcellular fractionation of hippocampal tissues of mock- and HSV-1-infected mice that revealed similar levels of cytoplasmic HDAC4 in both conditions, but a significant nuclear HDAC4 accumulation in hippocampal tissue from HSV-1-infected mice (+ 59%, p = 2.8 × 10^–3^ vs. mock-infected ones; Fig. 5A, B). To confirm the involvement of HSV-1 in HDAC4 nuclear accumulation we used the SH-SY5Y neuroblastoma cells transfected to express HDAC4 and infected or not with HSV-1. In basal condition, HDAC4 was distributed in both cytoplasmic and nuclear compartments, however 24 h after HSV-1 infection the amount localized in the nucleus significantly increased as assessed by biochemical fractionation (+ 75%, p = 2.8 × 10^–3^ vs. nuclear mock, Fig. S4A, B) and by confocal analysis (Mean Fluorescence Intensity [MFI] 29,445 ± 1395 vs. 13,108 ± 513, p = 2.9 × 10^–3^, Fig. S4C, D). Moreover, similarly to what observed in vivo, HSV-1-treated SH-SY5Y cells showed increased MeCP2 protein expression (+ 71%, p = 2.8 × 10^–3^, Fig. S4E, F). Based on these results, we reasoned that HDAC4 nuclear accumulation, induced by HSV-1, could promote the formation of a MeCP2/HDAC4 complex responsible for the reduced transcription of synaptic genes observed in HSV-1-infected mice. Co-immunoprecipitation (Co-IP) experiments revealed an increased association of HDAC4 with MeCP2 in hippocampal extracts of HSV-1-infected mice compared with mock ones (+ 59%, p = 2.8 × 10^–3^, Fig. 5C, D). This complex also included HDAC2 that was previously demonstrated to be a MeCP2 partner [35].

### HDAC4 regulates MeCP2 SUMOylation in HSV-1-infected mice and acts as gene co-repressor

The next step was to understand whether HDAC4 regulated MeCP2 function by acting as co-repressor on the promoter of target genes and/or by PTM. Indeed, MeCP2 DNA binding and repressive activity are controlled by several modifications including SUMOylation [37], a PTM able to modify the affinity, binding and stability of target proteins [38]. Since we recently demonstrated that HDAC4 acts as conjugating enzyme in the SUMOylation cascade [14], we investigated the role of HDAC4 in the SUMOylation of MeCP2. In preliminary experiments performed in SH-SY5Y cells we found that HDAC4 overexpression was associated with higher amounts of: (1) total SUMOylation (SUMO-1) and (2) MeCP2 level in comparison with control cells (+ 47% and + 41%, respectively p = 1.0 × 10^–4^ for both, HDAC4 vs. GFP-control, Fig. S4E–I). Following treatment with LMK235, a specific inhibitor of HDAC4, their expression returned to control values (p = 4.6 × 10^–3^ for SUMO-1 and p = 4.6 × 10^–4^ for MeCP2, HDAC4 vs. HDAC4 + LMK235, Fig. S4G–I), suggesting a possible contribution of HDAC4 to MeCP2 stability. Next, we investigated by Co-IP the level of SUMO-1-MeCP2 in cells expressing HDAC4 and we found that it was higher than in control cells (+ 121% p = 2.9 × 10^–3^ vs. mock; Fig. S4J–L). Similarly, we discovered that the SUMO-1-MeCP2 levels were greater in hippocampal tissues of HSV-1-infected mice than in mock-infected animals (+ 45%, p = 7.9 × 10^–4^, Fig. 5E, F).

Finally, to address the contribution of HDAC4 as MeCP2 co-repressor in gene regulation we performed a chromatin immunoprecipitation (ChIP) analysis evaluating HDAC4 recruitment on the promoters of some genes that we found downregulated in HSV-1-infected mice: the immediate early gene *c-fos* and the presynaptic gene *syn1* (Table 1 and Fig. 2G). The ChIP assay revealed that HDAC4 was enriched on the promoters of *c-fos*, and *syn1* in HSV-1-infected mice compared with controls (+ 3.9 and + 2.5 folds, respectively, p = 2.9 × 10^–3^ for both, Fig. 5G, H) confirming that HDAC4 may function as co-repressor on MeCP2 target genes.

## Discussion

Neuroinflammation, occurring in response to several types of insults, plays a key role in different CNS disorders, including neurodegenerative diseases, and has been associated with synapse dysfunction and cognitive decline in both animal models and patients [39]. In previous studies we reported that HSV-1 reactivations within the mouse CNS trigger neuroinflammation along with Aβ and pTau load in the hippocampus and neocortex [7]. However, accumulation of Aβ and pTau becomes relevant only after several cycles of virus replication whereas in the first rounds of reactivation the phenotype is primarily characterized by neuroinflammation (i.e., glial cell activation and increased levels of pro-inflammatory cytokines). Our results are in agreement with the literature reports indicating that IL-1β precedes amyloid deposition by promoting the processing of APP [40–42] and tau phosphorylation through aberrant activation of p38-MAPK leading to tangle formation [43–46]. Therefore, in the present study mice were subjected to 2TS to unveil the specific role played by neuroinflammation in synaptic and memory deficits of HSV-1-infected mice before the molecular hallmarks of AD exert their detrimental action.

Here we provide evidence that elevated levels of IL-1β, one of the key mediators of the immune response, negatively impinge on synapse structure and function, leading to memory deficits. In infected mice, IL-1-mediated alterations of hippocampal long-term potentiation and dendritic spines were associated with decreased expression of pre- and post-synaptic proteins and of synaptic-related genes which play key roles in critical brain functions ranging from consolidation of long-term memory to maintenance of synaptic density and dendritic architecture [47]. Notably, reduced gene expression might depend on the HSV-1-activated mechanisms of host mRNA degradation favoring virus survival [48]. Moreover, since HSV-1 genome is maintained in episomal form in the infected nuclei, we do not expect genetic modifications due to random virus integration. Previous studies [49] demonstrated that HSV-1 productive infection caused only impairment of non-homologous end joining pathway and DNA damage accumulation in primary neurons that in the long run may contribute to virus induced neurodegeneration.

Experimental findings support our contention that these adverse effects mainly depend on IL-1β elevation given that Anakinra, a pharmacological inhibitor of IL-1R, significantly restored gene expression in HSV-1-infected animals and ameliorated all molecular, structural and functional indices of synaptic plasticity. In HSV-1-infected mice, Anakinra failed to rescue memory soon after the TS, but its beneficial effects were evident during the following weeks. This finding suggests that the impact of HSV-1 replication on memory is complex and it is not limited to IL-1β downstream pathway only. However, when active virus replication ends and it is replaced by latency, the beneficial effects of IL-R blockade on memory were unveiled. We are tempted to speculate that when reactivated HSV-1 is actively replicating it can spread to brain regions that are important for learning and memory, there exerting a detrimental action on memory that is not fully due to IL-1R activation. Instead, when the virus has likely gone into latency, blockade of the molecular cascade downstream IL-1β is sufficient to rescue memory, an effect that was not observed in infected mice not treated with Anakinra. Notably, IL-1β may affect specific types and particular stages of memory in cognitive performance [50].

Our results are consistent with literature data demonstrating that Anakinra reverses neuroinflammation and related symptoms, including mental defects, in patients affected by a rare autoinflammatory disease exhibiting intellectual disability [51–53]. Similarly, treatments leading to reduced IL-1β levels (e.g., minocycline and FK506) counteracted neurodegenerative phenomenon in mice [54]. Our findings suggest that the contribution of IL-1β to synaptic defects relies on upregulation of the transcriptional repressor MeCP2 whose dysregulation has been revealed in various neurological and neurodevelopmental disorders [55]. Tomasoni et al. [33] demonstrated that elevated expression of MeCP2 mediates the alterations in spine morphogenesis, synaptic transmission and plasticity observed in the IL-1R8-KO mouse model exhibiting exaggerated signs of inflammations. Also, a significant increase of the complex Sin3a/HDAC1, recruited by MeCP2 to repress gene transcription, was reported in experimental models of HSV-1 infection [56].

Here we found that HSV-1-induced IL-1β increment determines a longer-lasting upregulation of MeCP2 that is paralleled by the downregulated expression of its target genes, such as *c-fos* and *syn1*, and decreased protein levels leading to synaptic and memory deficits. MeCP2 is reported as an intrinsically disordered protein that acquires a local secondary structures upon binding to other molecules [57]. As such, its target specificity and downstream function are determined by its molecular partners. We found that MeCP2 binds the chromatin remodeler HDAC4 whose alterations induce synaptic dysfunction and gene repression in experimental models of inflammation and AD [12–14]. In our mouse model of HSV-1 infection HDAC4 accumulated in the nuclei and triggered the formation of a MeCP2/HDAC4/HDAC2 molecular complex. The results of our ChIP experiments also showed that HDAC4 was recruited on the promoters of those plasticity-related genes that we found downregulated in HSV-1-infected mice. Of note, PTM of MeCP2 is another level of regulation that determines structural rearrangements, DNA binding, and stability. For example, MeCP2 SUMOylation modulates its repressive activity [38] and is crucial for the recruitment of HDAC1 and HDAC2 on the chromatin [58]. We have recently demonstrated that HDAC4, besides regulating protein acetylation, may function as E3 conjugating enzyme promoting protein SUMOylation [14, 59, 60]. Here we report that the association of HDAC4 and MeCP2 in HSV-1-infected mice results in the increase of MeCP2 SUMOylation which is consistent with its higher repressive activity and downregulation of target genes.

We also speculate that HDAC4-dependent MeCP2 SUMOylation, favoring its stability, may contribute to the sustained protein levels of MeCP2 observed when IL-1β levels drop. However, more research will be needed to fully address this intriguing issue.

Collectively, our findings provide novel evidence that following HSV-1 infection HDAC4 nuclear localization promotes MeCP2 SUMOylation and its interaction with HDAC members, thus unveiling a new pathogenic mechanism triggered by IL-1β affecting chromatin remodeling and gene expression. These results support the view that in HSV-1-infected mice neuroinflammation activated by repeated viral reactivations in the brain plays a prominent role in synaptic failure, especially at early phases of neurodegeneration when accumulation of Aβ and pTau within the brain is still limited.

### Supplementary Information

Below is the link to the electronic supplementary material.

Supplementary file1 (DOCX 2151 KB)

## Data Availability

The data used and /or analyzed during the current study are available from the corresponding author on reasonable request.

## Abbreviations

AD: Alzheimer’s disease
HDAC4: Histone deacetylase 4
HSV-1: Herpes simplex virus type-1
IL-1β: Interleukin 1β
IL-1R: Interleukin 1 receptor
iNOS: Inducible nitric oxide synthase
MeCP2: Methyl CpG binding protein 2
TSs: Thermal stresses
