# Supplementary material for: Interleukin 1β triggers synaptic and memory deficits in Herpes simplex virus type-1-infected mice by downregulating the expression of synaptic plasticity-related genes via the epigenetic MeCP2/HDAC4 complex
Source: Cell Mol Life Sci. 2023 Jun 1;80(6):172. doi: 10.1007/s00018-023-04817-5 (PMC10234878; doi:10.1007/s00018-023-04817-5)
Supplement: Supplementary file 1 — Supplementary file1 (DOCX 2151 KB) [file 18_2023_4817_MOESM1_ESM.docx]

**Supporting Information for**

**Interleukin 1β triggers synaptic and memory deficits in Herpes simplex virus type-1-infected mice by downregulating the expression of synaptic plasticity-related genes via the epigenetic MeCP2/HDAC4 complex**

Domenica Donatella Li Puma^#1,2^, Claudia Colussi^#2,3^, Bruno Bandiera^1^, Giulia Puliatti^1^, Marco Rinaudo^1,2^, Sara Cocco^1^, Fabiola Paciello^1,2^, Agnese Re^1^, Cristian Ripoli^1,2^, Giovanna De Chiara^4^, Alessia Bertozzi^1,3^, Anna Teresa Palamara^5,6^, Roberto Piacentini*^1,2^, Claudio Grassi^1,2^

^1^Department of Neuroscience, Università Cattolica del Sacro Cuore, 00168 Rome, Italy.

^2^Fondazione Policlinico Universitario A. Gemelli IRCCS, 00168 Rome, Italy.

^3^Department of Engineering, Istituto di Analisi dei Sistemi ed Informatica "Antonio Ruberti", National Research Council, Rome 00185, Italy.

^4^Institute of Translational Pharmacology, National Research Council (CNR), 00133 Rome, Italy.

^5^Department of Infectious Diseases, Istituto Superiore di Sanità, 00161 Rome, Italy.

^6^Department of Public Health and Infectious Diseases, Sapienza University of Rome, Laboratory affiliated to Istituto Pasteur Italia - Cenci Bolognetti Foundation, 00185 Rome, Italy.

# contributed equally.

***Corresponding author**: Roberto Piacentini, PhD

Department of Neuroscience, Università Cattolica del Sacro Cuore, 00168 Rome, Italy

Fondazione Policlinico Universitario A. Gemelli IRCCS, 00168 Rome, Italy

Phone number: (+39) 0630154966

E-mail: roberto.piacentini@unicatt.it

**Materials and methods**

**Mouse Synaptic Plasticity RT2 Profiler PCR Array**

RT^2^ Profiler PCR Array Mouse Synaptic Plasticity kit (Qiagen, Cat# PAMM-126ZA-6) was chosen because it includes diverse genes important in the mouse synaptic plasticity, including Immediate-Early Response (n = 30), Late Response (n = 2), Long Term Potentiation (n = 28), Long Term Depression (n = 21), Cell Adhesion (n = 9), Extracellular Matrix & Proteolytic Processing (n = 5), CREB Cofactors (n = 10), Neuronal Receptors (n = 19), Postsynaptic Density (n = 15), as well as other genes involved in the synaptic plasticity (n = 3). The array was performed in three independent sets of experiments. Relative abundance of each mRNA species was assessed using RT2 SYBR Green ROX qPCR Mastermix (Qiagen, Cat # 330524) and aliquoted in equal volumes (25 µl) to each well of the real-time PCR arrays. As indicated by the manufacturer, the real-time PCR cycling program was run on Applied Biosystem 7500 qRT-PCR thermal cycler. The threshold and baseline were set manually according to the manufacturer's instructions. The threshold cycle (Ct) data were uploaded into the data analysis template on the manufacturer's website. The relative expression of each gene in mock- and infected mice was calculated using ΔΔCT method with five housekeeping genes and compared with the expression in control mice.

**Immunohistochemistry and Image Analysis**

Mice were deeply anesthetized with ketamine and xylazine and were transcardially perfused with PBS (0.1 M, pH 7.4) followed by 4% paraformaldehyde. Brains were collected, post-fixed overnight at 4 °C in paraformaldehyde, and then transferred to a solution of 30% sucrose in 0.1M PBS. Coronal sections (40 μm) were then obtained using a vibratome (VT1000S, Leica Microsystems, GmbH, Wetzlar, Germany). The following day slices were incubated with blocking buffer containing PBS with 1% bovine serum albumin (BSA), 10% normal goat serum (NGS, Sigma), and 0.5% Triton-X 100 for 2h at RT. Then, slices were treated with the primary antibodies (see table S3) diluted in PBS containing 1% BSA, 5% NGS, and 0.3% Triton-X100 for 48 h at 4°C. Then, slices were incubated with the appropriate secondary antibody diluted in PBS for 90 min at RT. Nuclei were then counterstained with 4’,6-diamidino-2-phenylindole (DAPI, 1:500, Thermo). Finally, slices were coverslipped with ProLong Gold antifade reagent (Thermo). Confocal stacks made of images (1,024 × 1,024 pixels) were acquired at 40× magnification with a confocal laser scanning system (TCS-SP5, Leica) and an oil-immersion objective. Fluorescent dyes were excited with lasers at 405, 488 and 560 nm. IF for synaptophysin, synapsin-1 was quantified by drawing regions of interest (ROIs) on CA1 and CA3 pyramidal neurons and/or area covering the Schaffer collaterals, i.e. near the CA3 and under the CA1 region, identified by DAPI or NeuN immunoreactivity. In every studied condition, negative controls were obtained by omitting the primary antibodies. The operator was blinded to the study condition.

**Primary hippocampal neuron cultures and HSV-1 infection**

Primary cultures of hippocampal neurons were obtained from E18 C57BL/6 mice as previously described [19]. After 12 days in vitro, 1×10^6^ cells were infected with at a multiplicity of infection (MOI) of 5 and then incubated for 1 hour at 37 °C, a time interval required for virus binding and entry into host cells (adsorption period). Anakinra (1.49 mg/ml) was added to the culture medium, 1 hour before, during and after the adsorption time. Twenty-four hours post infection, the supernatants of infected cells were subjected to standard plaque assay to quantify virus production [17].

**SH-SY5Y culture and treatments**

SH-SY5Y neuroblastoma cell line was grown in DMEM/Ham’s F12 medium supplemented with 10% inactivated fetal bovine serum, 1% penicillin-streptomycin antibiotic mixture (Sigma-Aldrich) and 2 mM L-glutamine (Thermo). Transient overexpression of GFP-HDAC4 or GFP (Addgene, Watertown, MA USA), was achieved by transfection of plasmid DNA with PEIMax (Polyethylenimine Hydrochloride, Polysciences) according to manufacturer’s instructions. The day after transfection the cells were infected with HSV-1 (MOI 1) and processed, along with mock controls, for IF or biochemical fractionation or immunoprecipitation. Cells expressing GFP-HDAC4 or GFP were treated with the HDAC4 specific inhibitor LMK235 (40mM, 24 h) and then processed by WB for MeCP2 protein expression or total SUMOylation identified as multiple bands corresponding to post-translationally SUMO-modified proteins.

**Confocal analysis of HDAC4 nuclear localization**

SHSY-5 cells expressing GFP-HDAC4WT and infected with HSV-1 were fixed in 4% paraformaldehyde for 10 min, permeabilized for 10 min in 0.3% Triton-X100 in PBS and labelled with DAPI for the detection of the nuclei. Samples were analyzed with a confocal laser scanning system (Nikon A1MP equipped with 20×-40×-60×-100× objectives). Confocal settings were the same for all examined samples to compare fluorescence intensities. 3D images of cells, deriving from reconstruction of Z stacks, were used to calculate the Mean Fluorescence Intensity (MFI) by Image J software. Signals from single region of interests (nuclei) were used to calculate the average values for fluorescence intensity. Ranges of 50-80 nuclei for each experimental condition were analyzed (4 biological replicates).

**Figures and legends**

**
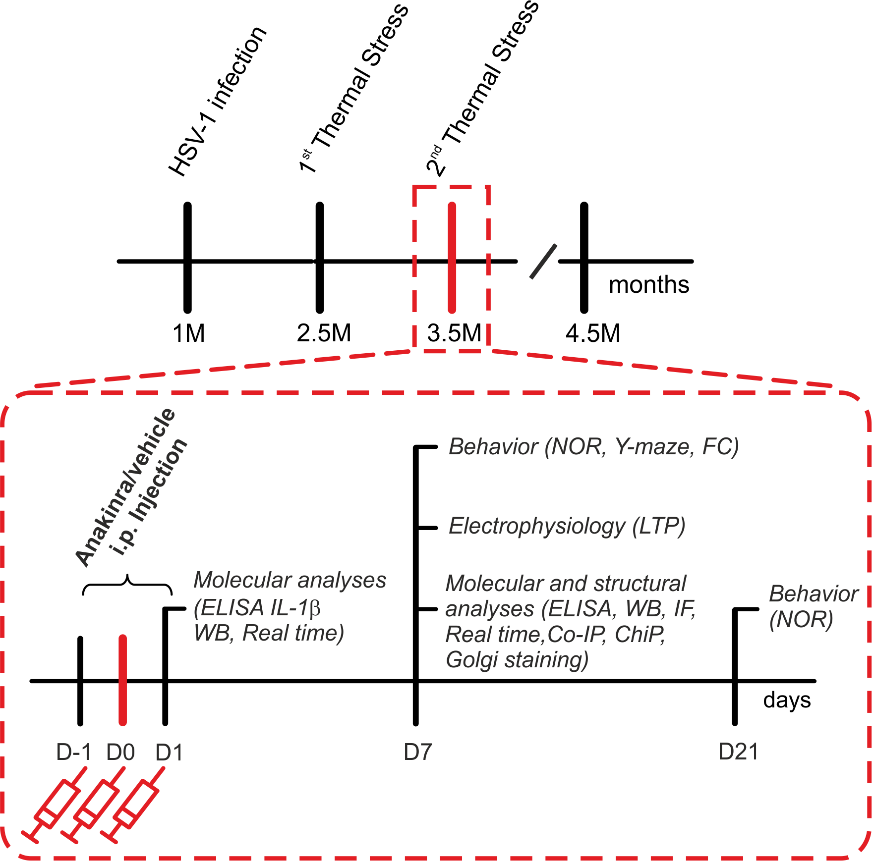
**

**Fig. S1. Schematic representation of the experimental design and timeline.**

The mouse model of recurrent HSV-1 infection was established in 1-month-old C57BL/6 mice inoculated with HSV-1 via lip scarification. After 6 weeks (2.5 months) and 10 weeks (3.5 months) from the primary infection virus was reactivated in the brain by exposing mice to hyperthermia. Close to the 2^nd^ thermal stress (TS), animals received an intraperitoneal injection of either Anakinra (30 mg/Kg) or vehicle for 3 consecutive days [the day before TS (D-1), the day of TS (D0), and the day after TS (D1)] and studied at D1, D7 and D21.


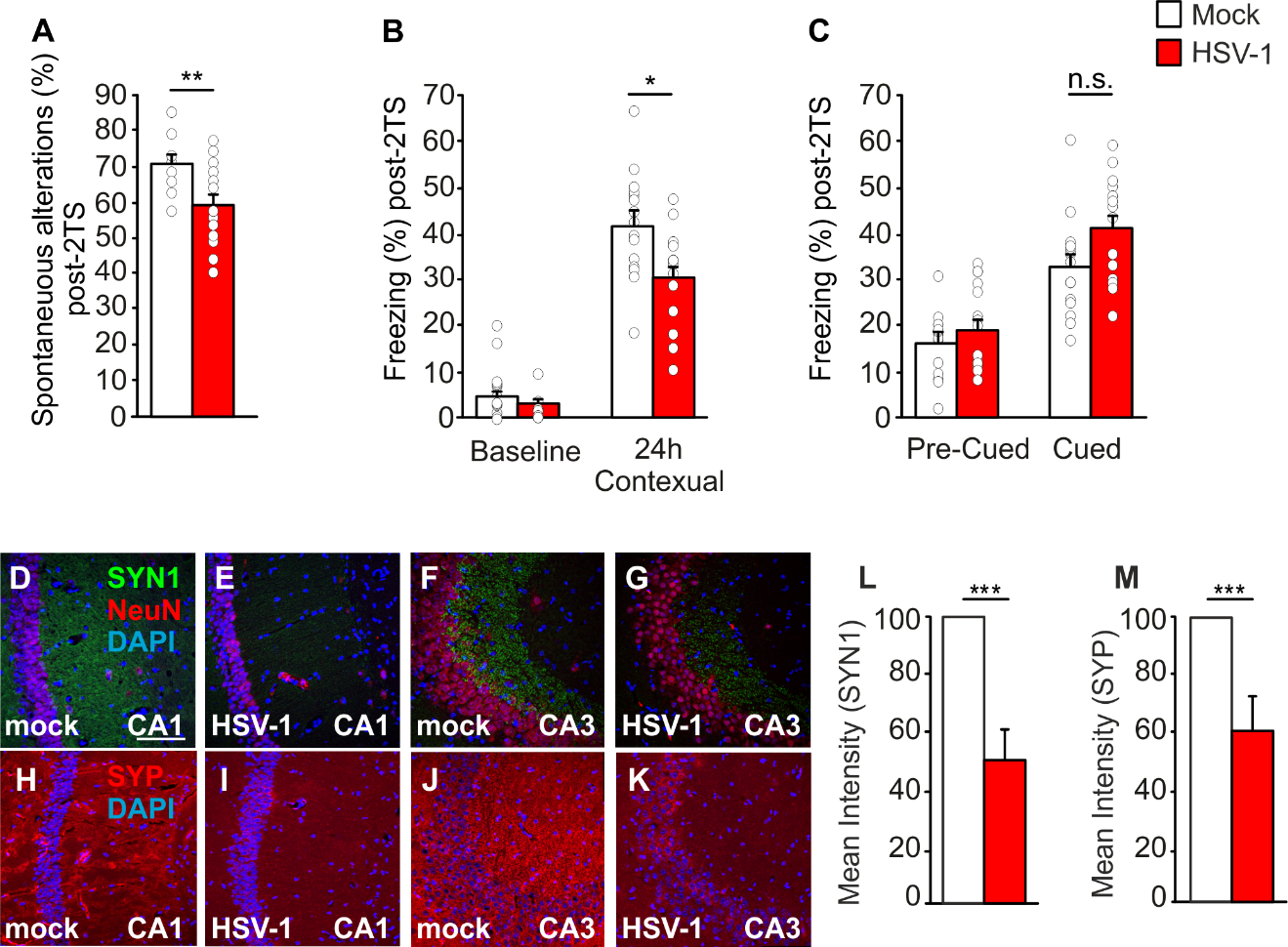


**Fig. S2. Recurrent HSV-1 reactivations within the brain induces memory and synaptic impairment**

**(A, B**) Bar graphs summarizing the percentage of (A) spontaneous alternation behavior in the Y-maze in mock- and HSV-1-infected mice (n=10 and 15, respectively) and (B) freezing during the baseline period of training and 24 hours after the electric shock, in mock- and HSV-1-infected mice subjected to 2TS (n=15 and 14 mice, respectively); **(C)** Freezing response before (pre) and after the auditory cue among the same groups of animals described in B; **(D-K)** Confocal images from representative coronal hippocampal sections of mock- and HSV-1-infected mice undergone 2TS showing the immunoreactivity for SYN1 (D-G) and SYP (H-K) in CA1 and CA3 hippocampal regions; **(L, M)** Bar graphs showing the mean intensity of SYN1 (L) and SYP (M) immunoreactivity in mock- and HSV-1-infected mice (n=3 mice for each condition). *p<0.05, **p<0.01 and ***p<0.005 vs. mock, assessed by two-way ANOVA and Newman-Keuls post-hoc (panels A, B, C) and by Mann-Whitney Rank Sum Test (panels L, M). n.s.: not significant difference. Scale bar 100 µm.

**
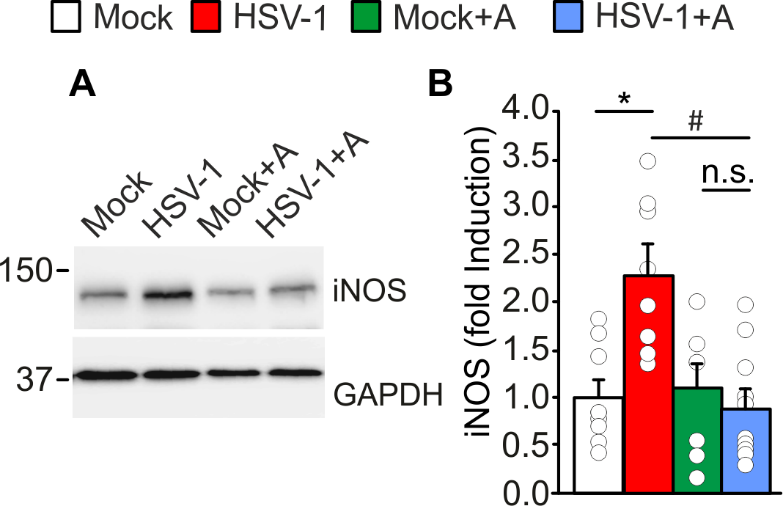
**

**Fig. S3. The IL- 1R antagonist, Anakinra, prevents the increased expression of iNOS in HSV-1-infected mice**

**(A)** Representative WB images of iNOS expression in mock- and HSV-1-infected animals treated and untreated with Anakinra (mock+A and HSV-1 +A, respectively); **(B)** Densitometric analysis of WB represented in A analyzing n=9 mock; n=8 HSV-1; n=8 mock+Anakinra and n=10 HSV-1+Anakinra. *p<0.05 vs. mock, #p<0.05 vs. HSV-1, assessed by Mann-Whitney Rank Sum Test. n.s. means not significant difference.

**
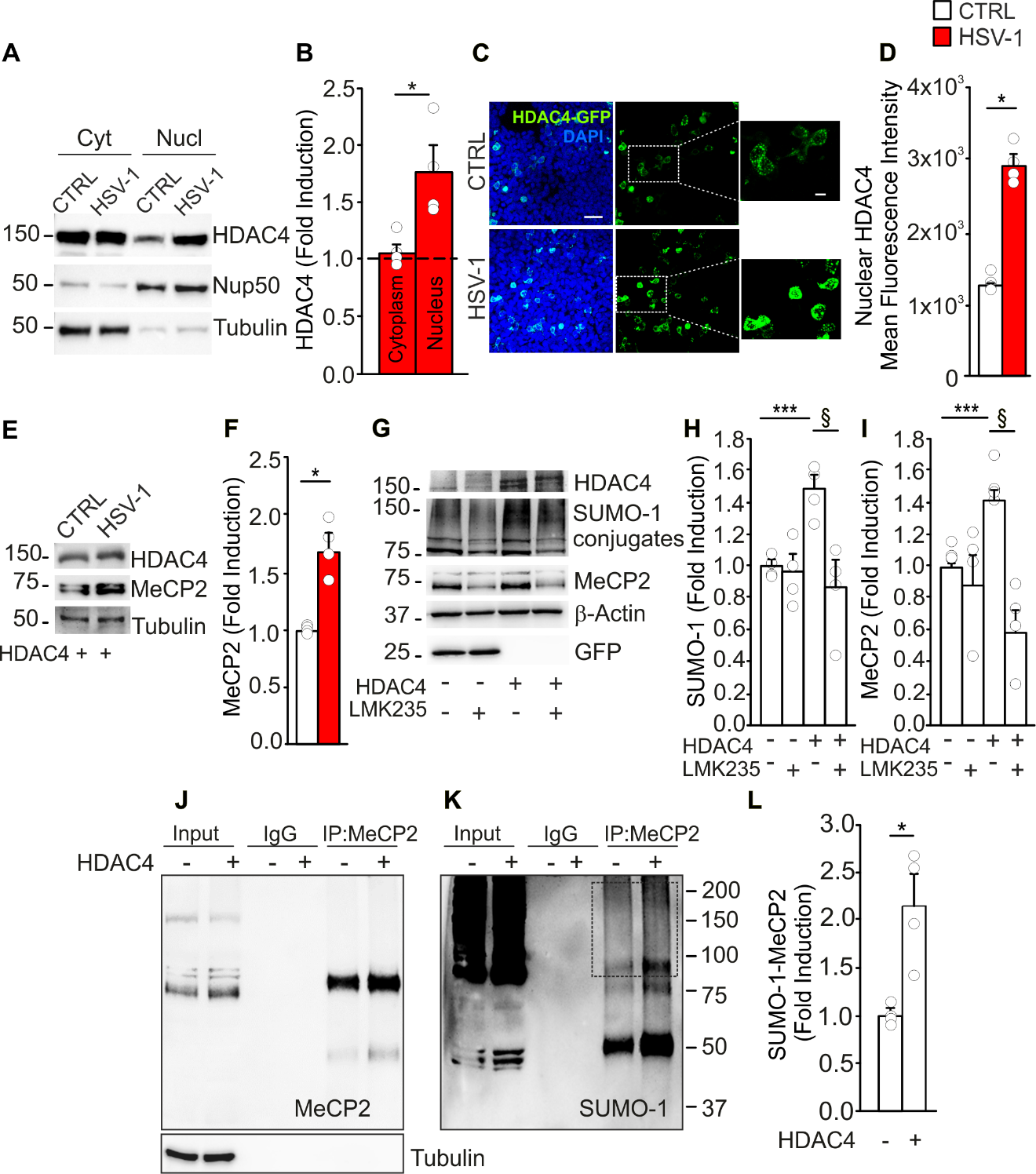
**

**Fig. S4. Infected SH-SY5Y exhibited increased nuclear accumulation of HDAC4 and MeCP2-SUMOylation**

**(A, B)** Nuclear and cytoplasmic fractionation of SH-SY5Y cells, expressing HDAC4 and infected with HSV-1, analyzed by WB and relative densitometric analyses (n=4 independent experiments). Nup50 was used as nuclear loading control. Dotted line indicates the levels of nuclear and cytoplasmic HDAC4 in controls; **(C)** Confocal images of SH-SY5Y cells overexpressing HDAC4-GFP construct (green) in mock- (upper panels) and infected cells (lower panels); **(D)** Bar graph showing the MFI of nuclear HDAC4 in infected cells expressing GFP-HDAC4 in control condition and after HSV-1 infection (n=4 independent experiments); **(E,F)** representative WB of MeCP2 protein level (E) and its quantification (F) in SH-SY5Y expressing HDAC4 (n=4 independent experiments); **(G-I)** total SUMOylation (G, H) and MeCP2 protein (G, I) levels analyzed by WB in SH-SY5Y expressing HDAC4 or GFP and treated with or without LMK235 and the relative densitometry (n=4 independent experiments); **(J-L)** Analysis of MeCP2 (J) and its SUMOylation (K) levels in SH-SY5Y cells overexpressing or not HDAC4 construct and relative quantification (L, n=4 independent experiments). The dotted box indicates the pattern of SUMO-1-MeCP2 modified proteins. *p<0.05 and ***p<0.005 vs. ctrl, and §p<0.05 vs. HDAC4+LMK235 assessed by Mann-Whitney Rank Sum Test. Scale bars: 50 and 5µm.

**Table S1: Antibodies**

| **Primary Antibody** | **Host and dilution** | **Catalogue reference** |
| --- | --- | --- |
| α-β Actin | Rabbit 1:2000 | Abcam #8227 |
| α-GAPDH | Mouse 1:1000 | Abcam #9484 |
| α-GFP | Rabbit 1:1000 | Thermo Fisher #G10362 |
| α-GluA1(D4N9V) | Mouse 1:1000 | Millipore #2263 |
| α-HDAC2 | Mouse 1:1000 | Santa Cruz #sc-7899 |
| α-HDAC4 | Rabbit 1:1000 | Abcam #12172 |
| α-iNOS | Rabbit 1:500 | Abcam #3523 |
| α-MeCP2 | Rabbit 1:1000 | Sigma #M9317 |
| α -NeuN | Mouse 1:300 | Millipore #MAB337 |
| α-NMDAR2B | Mouse 1:1000 | BD Bioscience #610417 |
| α -Nup50 | Rabbit 1:1000 | Abcam #85915 |
| α-PSD95 (D27E11) | Rabbit 1:1000 | Cell Signaling #3450 |
| α-Synapsin | Rabbit 1:1000 | Cell Signaling #5297 |
| α-Synaptophysin | Mouse 1:1000 | Abcam #8049 |
| α-SUMO1 | Mouse 1:1000 | Cytoskeleton #ASM01-FS |
| α-Tubulin | Mouse 1:4000 | Abcam #7291 |
| Alexa Fluor-488 anti-rabbit | Donkey 1:500 | Thermo Fisher #A21206 |
| Alexa Fluor-546 anti-mouse | Goat 1:500 | Thermo Fisher #A11003 |

**Table S2: Primer sequences used for Real Time PCR analysis.**

| **Genes** | **Primers sequences** |
| --- | --- |
| *Mecp2* | FW 5’-CTCCATAAAAATACAGACTCACCAGT-3’ |
|  | RW 5’-CTTAAACTTCAGTGGCTTGTCT-3’ |
| *Syn1* | FW 5’-CAGGGTCAAGGCCGCCAGTC-3’ |
|  | RW 5’-CACATCCTGGCTGGGTTTCTG-3’ |
| *C-fos (Chip)* | FW 5’-TCCAGTTCCGCCCAGTGA-3’ |
|  | RW 5’-TCAGCTGGCGCCTTTATAGAA-3’ |
| *Syn1 (Chip)* | FW 5’-TATCCCCAAAATGCCTTCAA-3’ |
|  | RW 5’-TTGCCCTTCCCAGATACTTG-3’ |

Abbreviations: FW, forward; RV, reverse
